# Ascorbic acid prevents stress-induced hypercoagulability in overweight and obese individuals

**DOI:** 10.1038/s41598-024-53794-7

**Published:** 2024-02-07

**Authors:** Helena N. M. Rocha, Larissa L. Velasco, Gabriel M. S. Batista, Amanda S. Storch, Vinicius P. Garcia, Gabriel F. Teixeira, Juliana Mentzinger, Antonio C. L. da Nóbrega, Natália G. Rocha

**Affiliations:** 1 Laboratory of Exercise Sciences, Department of Physiology and Pharmacology, Fluminense Federal University, Rua Alameda Barros Terra, Sala 110, São Domingos, Niterói, RJ 24.020-150 Brazil; 2 Laboratory of Integrative Cardiometabology, Department of Physiology and Pharmacology, Fluminense Federal University, Rua Alameda Barros Terra, Sala 110, São Domingos, Niterói, Rio de Janeiro Brazil; 3 National Institute of Science and Technology (INCT) - Physical (In)Activity and Exercise, National Council for Scientific and Technological Development (CNPq), Rua Alameda Barros Terra, Sala 110, São Domingos, Niterói, Rio de Janeiro Brazil

**Keywords:** Coagulation, Obesity, Ascorbic acid, Mental stress, Nitric oxide, Cardiovascular diseases, Platelets

## Abstract

Ascorbic acid (AA) may contribute to restoring hemostatic balance after mental stress (MS) in overweight/obese adults. We aimed to determine the effects of AA administration on hemostatic responses to MS in overweight/obese men. Fourteen overweight/obesity men (27 ± 7 years; BMI: 29.7 ± 2.6 kg m^−2^) performed the Stroop color-word stress task for 5 min after non-simultaneous infusion of placebo (PL, 0.9% NaCl) and AA (3 g). Blood was collected at baseline, during MS, and 60 min after MS to measure: activated partial thromboplastin time, prothrombin time, and fibrinogen concentration, by coagulometer; platelet-derived microvesicles (PMV, mv/μL), by flow cytometry; nitrite (μM), by chemiluminescence. In PL session, MS led to decreases in PTs (stress, *p* = 0.03; 60 min, *p* < 0.001), PT-INR (stress, *p* < 0.001; 60 min, *p* < 0.01), aPTTs (60 min, *p* = 0.03), aPTT ratio (60 min, *p* = 0.04) and fibrinogen (60 min, *p* = 0.04), while increased PT activity (60 min, *p* = 0.01) when compared to baseline. Furthermore, AA increased PTs (60 min, *p* < 0.001), PT-INR (60 min, *p* = 0.03) and decreased PT activity (60 min, *p* < 0.001) and fibrinogen (stress, *p* = 0.04) when compared to PL. Nitrite was increased in response to stress during AA session (*p* < 0.001 vs PL). There was no difference in PMV. Ascorbic acid prevented the impaired hemostatic profile and improved nitrite response to stress in the overweight and obese adults.

## Introduction

Psychological or mental stress induces a transient endothelial dysfunction and prothrombotic phenotype^1,2^. The hypercoagulability response to stress protects a healthy organism from bleeding during fight-or-flight conditions. However, chronic stress exposure enhances the risk of acute myocardial infarction and cardiovascular mortality by two times in adults at increased cardiometabolic risk, such as overweight or obese ones^3,4^.

Besides its role as vasodilator, nitric oxide (NO) may reduce the risk of atherothrombotic events^5,6^. During vascular damage, platelets are activated, and hemostatic responses are amplified to restore hemostatic balance with an occlusion plug formation^7,8^. Also, activated platelets can release platelet-derived microvesicles (PMV), biomarkers of endothelium damage^9,10^ with intercellular signaling functions^11^. NO regulates platelet function in both autocrine and paracrine manners, increasing cyclic GMP and reducing platelets adhesion and aggregation^6^.

Studies have demonstrated that sympathetic drive during stressful conditions activates the renin–angiotensin–aldosterone system^12,13^, increasing reactive oxygen species (ROS) by NADPH complex, ROS scavenger NO and increase peroxynitrite (ONOO^-^), which stimulates coagulation cascade and a hypercoagulability condition.

Considering that stress exposure and overweight/obesity are independent cardiovascular risk factors and are present in a large portion of the world's population; it is important to understand the underlying mechanisms that explain the increased atherothrombotic events when both conditions are combined^14^. Hence, our purpose was to test the hypothesis whether the administration of ascorbic acid (AA), a potent antioxidant^15^, may contribute to reducing prothrombotic factors, even under stressful situations in overweight/obese individuals.

## Materials and methods

Fourteen overweight and obese men [Body mass index (BMI) between 25.0 and 34.9 kg m^−2^^16^] were selected by print and electronic media based on the following inclusion criteria: absence of any chronic diseases (hypertension, diabetes, chronic kidney disease, heart failure, among others, except obesity), hemostatic impairments and musculoskeletal abnormalities; do not practice physical activity regularly (exercise lasting ≥ 30 min, 3 times a week); do not make regular use of medications and/or antioxidants. The present study’s protocol was approved by the Institution’s Ethics Committee (CAAE: 76594217.0.0000.5243) and followed the Declaration of Helsinki’s ethical standards. All participants were informed about the study’s protocol procedures and signed a consent form.

Three non-consecutive visits to the Laboratory of Exercise Sciences (LACE) at Fluminense Federal University were requested to carry out this study. On the first visit, anthropometric measures and body composition were analyzed by bioimpedance (RJL Systems Body Composition software—Clinton Township, MI, United States). Also, venous blood samples were collected after a 12-h fasting period, for the following biochemical tests: fasting glycemia, total cholesterol and triglycerides (dry chemistry; Vitros, Ortho-Clinical Diagnostics, New Jersey, United States).

The two following visits were randomized to experimental sessions with placebo (PL) or AA administration.

### Experimental sessions

Visits to the laboratory took place in the morning, in an environment with controlled temperature (22–24 °C). All participants were instructed not to ingest alcohol, caffeine and food with high concentration of antioxidants, as well as not to perform physical exercises in the 48 h prior to the experimental sessions. Subjects ingested a light meal three hours before the experimental sessions. Prior to experimental protocol, electric bioimpedance was performed in order to measure fat mass of participants.

**Figure 1:**
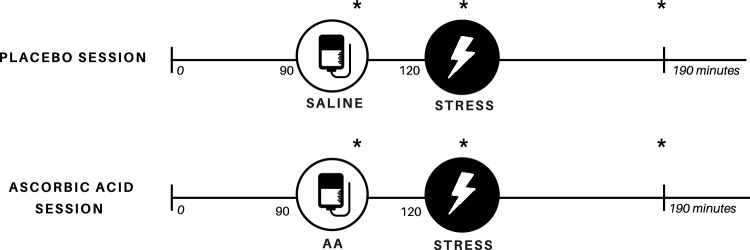
Experimental protocol. Asterisk indicate blood collection. AA, ascorbic acid.

It is a randomized, single-blinded, placebo-controlled and crossover study. The experimental protocol was characterized by two sessions with either an intravenous administration of ascorbic acid (AA) or placebo (PL) in non-consecutive days, with an interval of at least seven days among them. All participants were subjected to both AA and PL administrations. In the beginning of each session, baseline hemodynamic and anthropometric variables were measured. Then, participants were instrumentalized at supine position, a catheter was placed in antecubital vein for infusion and blood sampling. During AA session, 3 g of AA diluted in 500 mL of saline solution was venous infused during 30 min; whereas in the PL session, only 500 mL saline venous infusion was used. Hemodynamic variables were measured throughout the experimental sessions. Venous blood samples were collected for hemostatic biomarkers and nitrite determination at baseline, during the last minute of the stress task, and 60 min after stress protocol. This study’s experimental design is represented in Fig. 1.

### Hemodynamic variables

Hemodynamic variables were evaluated throughout the experimental protocol. Blood pressure was continuously checked, beat by beat, by means of a non-invasive system (Finometer, Finapres Measurement Systems, Arnhem, Netherlands). Heart rate was obtained by electrocardiographic recording. All signals were obtained at a frequency of 1000 Hz, integrated, and digitized through the PowerLab data acquisition system (ADInstruments, Sydney, Australia) and analyzed by LabChart 8 software (ADInstruments, Sydney, Australia).

### Stress task

**Figure 2:**
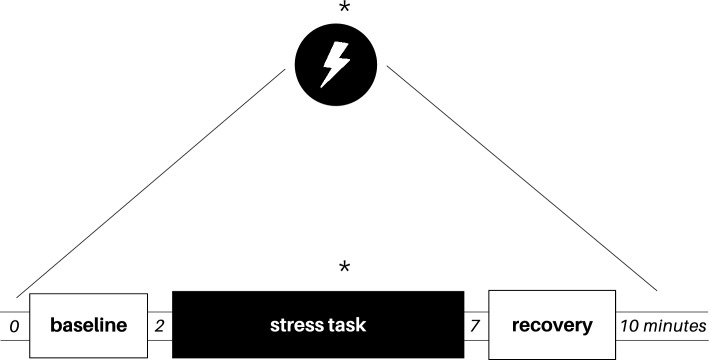
Experimental design of stress task protocol. Asterisks indicate blood collection.

Stress was induced by a modified version of the Stroop color-word test^17^, in which the participants received three color information in a slideshow that changed every two seconds and were instructed to read quickly and aloud, the font color of the written word, successively. At the same time, the participants experienced a hearing conflict with voices that repeat, in a headset, colors different from those observed by the participant. Stress task was divided into two minutes of baseline measurements, five minutes of task induced by Stroop color-word test and three minutes of recovery, in which the participants rested in silence after the completion of the test. The level of perceptible/subjective stress was evaluated after the task with a scale from zero to four, in which: 0 = non-stressful, 1 = slightly stressful, 2 = stressful, 3 = very stressful and 4 = extremely stressful. Stress task protocol experimental design is represented in Fig. 2.

### Hemostatic variables

#### Prothrombin time

Prothrombin time (PT) analysis was performed using HumaClot Jr. coagulometer (Human GmbH Max; Wiesbaden, Germany). The test was used to determine the activity of the extrinsic and common coagulation pathways by adding a source of factor (thromboplastin), which activates factor VII, and calcium. Plasma recalcification in the presence of thromboplastin generates activated factor Xa and, consequently, thrombin formation and, subsequently, an insoluble fibrin clot. In the present study, 200 μL of thromboplastin was added to the plasma collected in a citrate tube. The result was expressed in seconds, percentage activity in relation to normal (%act) and international normalized ratio (INR)^18^.

#### Activated partial thromboplastin time

Activated partial thromboplastin time (aPTT) analysis was also done by using HumaClot Jr. coagulometer (Human GmbH Max; Wiesbaden, Germany), and consists of determining the clotting time of the treated plasma, after the addition of reagents containing a plasma activator (ellagic acid) and phospholipids, which will act as platelet substitutes. The mixture is incubated for activation, and then recalcified with calcium chloride (CaCl_2_) triggering the intrinsic pathway coagulation mechanism. This step is timed until the formation of the clot^18^, and the results were expressed in seconds and ratio (test result divided by a control plasma’s result).

#### Fibrinogen concentration

The fibrinogen levels were measured through citrated plasma using the HumaClot Jr. coagulometer (Human GmbH Max; Wiesbaden, GER). Briefly, 50μL of human thrombin were added to 100 μL of plasma previously diluted with a buffered saline solution of Imidazole (0.05 mol/L), and incubated at 37 °C. The clotting time is inversely proportional to the concentration of fibrinogen in the sample.

#### Platelet-derived microvesicles

Venous blood samples were collected in sodium citrate tubes and centrifuged at 2000 *g* for 15 min at room temperature. This centrifugation procedure was repeated twice to obtain platelet-poor plasma. Then, plasma was collected and stored at − 80 °C. For analysis, 25 μL of plasma was incubated with 5 μL of CD41-APC monoclonal antibody for 30 min, at 4 °C, in the dark. Samples were diluted in 225 μL of phosphate-saline buffer (PBS) before flow cytometry analysis. TruCount tubes (BD Biosciences, Franklin Lakes, NJ, United States) were used for absolute microparticle count per μL of plasma, using the formula provided by manufacturer: (number of events acquired/absolute number of TruCount beads) X (total number of TruCount beads per test/total sample volume). All samples were analyzed using a flow cytometer (BD FACSVerse; BD Biosciences; Franklin Lakes, NJ, United States)^19^.

### Nitrite concentration

Plasma nitric oxide levels were indirectly evaluated from nitrite concentration in the participants’ samples using NO Analyzer (NOA, Nitric Oxide Analyzer Sievers), model 280i (GE Analytical Instruments, Colorado, United States). The method is based on measurement of nitrite resulting from the oxidation of NO. Initially, a reducing agent (1% wt/vol of NaI or KI in acetic acid) is used for conversion of nitrite into NO. Later on, 20μL of deproteinized human serum in 5 mL of acidified tri-iodide solution is injected into the sealed glass chamber of the analyzer. NO gas reaction with ozone is detected by chemiluminescence. Concentrations were determined by interpolation of a nitrite standard curve (Sigma-Aldrich, Missouri, United States)^20^.

### Statistical analysis

Sample size calculation was previously performed considering a statistical power of 0.8 and an alpha error of 0.05. A minimum sample size required for PT INR and nitrite responses to stress (main outcomes) were 11 and 10 individuals, respectively. The Shapiro–Wilk test was used to verify the normality in the distribution of the variables and homoscedasticity was performed by the Levene test. Data are represented as the mean ± standard error of the mean. Repeated measures analysis of variance was used to compare hemostatic variables before, during and 60 min after stress between PL and AA sessions. Aside from hemodynamic variables, values were expressed as fold changes from baseline. Student's t-test or its respective non-parametric test was used to compare the hemostatic variables' response to stress (Δ, 60 min minus baseline). A *p*-value less than or equal to 0.05 was considered statistically significant. GraphPad Prism software (version 8.0, San Diego, California, USA) was used for analysis.

## Results

Fourteen participants aged 27 ± 2 years old and body mass index (BMI) averaged 29.7 ± 2.6 kg m^−2^ (fat mass: 31.8 ± 0.94%; glucose: 88 ± 2.34 mg/dL; total cholesterol: 178 ± 12.42 mg/dL; triglycerides: 119 ± 17.34 mg/dL) were considered in the sample. Hemodynamic variables at baseline, during and after stress task in PL and AA sessions are presented in Table 1. Stress task increased heart rate, systolic, diastolic, and mean blood pressures similarly in all sessions (*p* < 0.05 vs baseline) and returned to baseline levels during recovery (*p* < 0.05 vs stress). The level of perceived stress was assessed in both visits by showing the subject a table containing the aforementioned perceived stress scale. The average level of perceived stress for both sessions was 2 (stressful).

**Table 1:** Hemodynamic variables at baseline, during and at recovery of stress task in placebo and ascorbic acid sessions.

Variables	Placebo			Ascorbic acid		
	Baseline	Stress	Recovery	Baseline	Stress	Recovery
SBP (mmHg)	123 ± 3	136 ± 3*	124 ± 3^†^	124 ± 3	139 ± 2*	126 ± 3^†^
DBP (mmHg)	76 ± 2	88 ± 3*	77 ± 2^†^	79 ± 2	91 ± 2*	80 ± 2^†^
MAP (mmHg)	92 ± 2	104 ± 3*	93 ± 2^†^	94 ± 2	107 ± 2*	95 ± 2^†^
Heart rate (bpm)	62 ± 2	76 ± 3*	64 ± 2^†^	65 ± 3	76 ± 3*	65 ± 3^†^

Data are expressed as the mean ± standard error of the mean (SEM). SBP, systolic blood pressure; DBP, diastolic blood pressure; MAP, mean arterial pressure. *p < 0.05 versus baseline; ^†^*p* < 0.05 versus stress (n = 14).

### Hemostatic measurements

**Figure 3:**
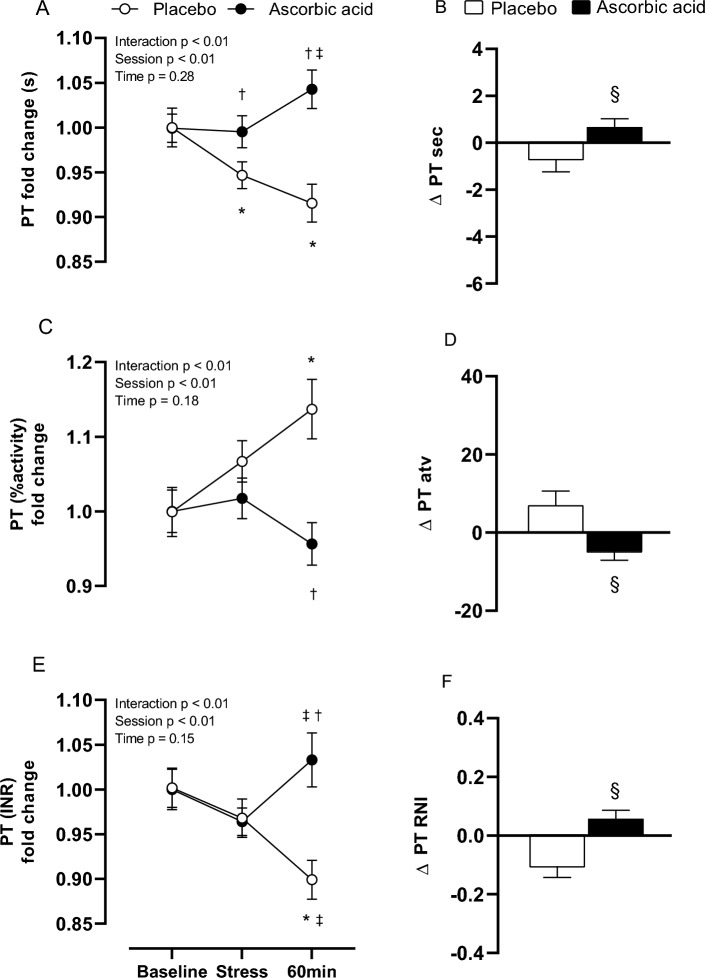
Prothrombin time (PT) in seconds, percentual activity and international normalized ratio (**A**, **C**, **E**) and their respective delta responses to stress (**B**, **D**, **F**) after placebo (PL) and ascorbic acid (AA) administration in overweight/obese men. Δ represents 60-min minus baseline. *p < 0.05 versus baseline; ^†^*p* < 0.05 versus placebo; ^‡^*p* < 0.05 versus stress; ^§^*p* < 0.05 versus placebo.

Stress induced a reduction in PT seconds (baseline, 1.00 ± 0.02 s; stress, 0.95 ± 0.01 s, *p* = 0.03; 60 min after stress, 0.92 ± 0.02 s, *p* < 0.001) and INR (baseline, 1.00 ± 0.02; stress, 0.97 ± 0.02, *p* < 0.001; 60 min after stress,0.90 ± 0.02, *p* < 0.01) as well as an increase of PT activity (baseline, 1.00 ± 0.03% vs 60 min after stress, 1.14 ± 0.04%, *p* < 0.001) at PL session. During AA session, PT seconds was higher during stress and 60 min after stress in comparison to placebo session (PL stress, 0.95 ± 0.01 s vs AA stress, 1.00 ± 0.02 s, *p* = 0.04; PL 60 min after stress, 0.92 ± 0.02 s vs AA 60 min after stress, 1.03 ± 0.03 s, *p* < 0.001). PT INR increased during AA session at 60 min after stress (stress, 0.97 ± 0.02 vs 60 min after stress, 1.03 ± 0.03, *p* = 0.03), which was higher than the same moment in the PL session (PL, 0.90 ± 0.02 vs 60 min after stress, 1.03 ± 0.03, *p* < 0.01). During AA session, PT activity was reduced in comparison to the placebo session at 60 min after stress (PL, 1.14 ± 0.04% vs AA, 0.96 ± 0.03%, *p* < 0.0001). In response to stress, both PT seconds (PL, Δ − 0.69 ± 2.02 s vs AA, Δ 0.66 ± 1.18 s, *p* = 0.003) and INR were increased during AA session (PL, Δ − 0.10 ± 0.14 vs AA, Δ 0.06 ± 0.10, *p* < 0.001) while PT activity presented a significant reduction (PL, Δ7.00 ± 13.62% vs AA, Δ − 4.81 ± 7.53%, *p* = 0.02) (Fig. 3).

**Figure 4:**
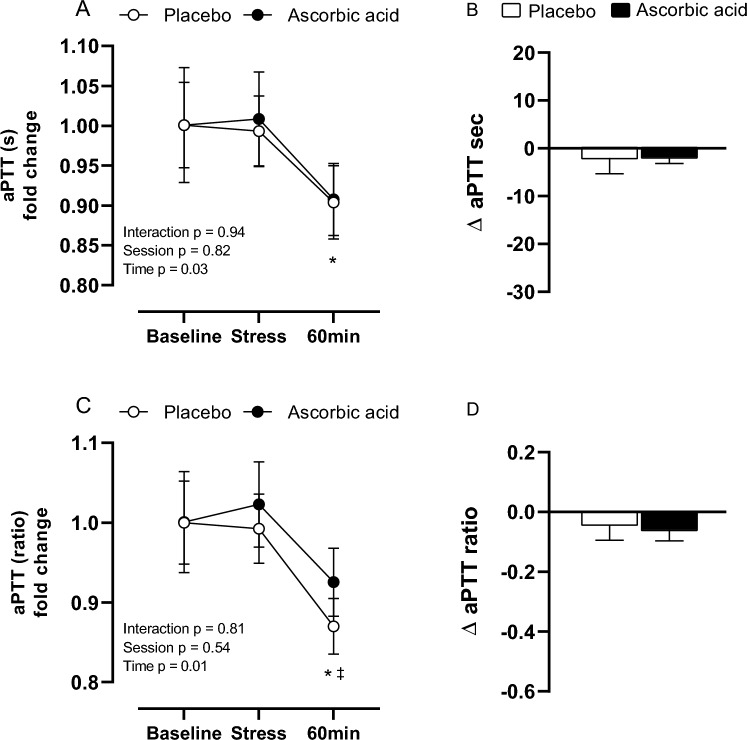
Activated partial thromboplastin time (aPTT) in seconds and ratio (**A**, **C**) and their respective delta responses to stress (**B**, **D**) after placebo (PL) and ascorbic acid (AA) administration in overweight/obese men. Δ represents 60-min minus baseline. *p < 0.05 versus baseline; ‡p < 0.05 versus stress.

There was a significant reduction in aPTT seconds (baseline, 1.00 ± 0.05 s vs 60 min after stress, 0.90 ± 0.05 s; *p* = 0.04) and a PTT ratio (baseline, 1.00 ± 0.05 s vs 60 min after stress, 0.87 ± 0.03 s, *p* = 0.03; stress, 0.99 ± 0.0 vs 60 min after stress, 0.87 ± 0.03 s, *p* = 0.04) in PL session. However, no differences were observed at AA session for neither aPTT seconds (baseline, 1.00 ± 0.07 s; stress, 1.01 ± 0.06 s; 60 min after stress, 0.90 ± 0.04 s) nor ratio (baseline, 0.93 ± 0.04 s; stress, 1.02 ± 0.05 s; 60 min after stress, 1.00 ± 0.06 s). Also, there were no differences in stress response for aPTT seconds (PL, Δ − 2.00 ± 11.36 s vs AA, Δ − 1.88 ± 4.42 s) or ratio (PL, Δ − 0.042 ± 0.18 s vs AA, Δ − 0.06 ± 0.12 s) (Fig. 4).

**Figure 5:**
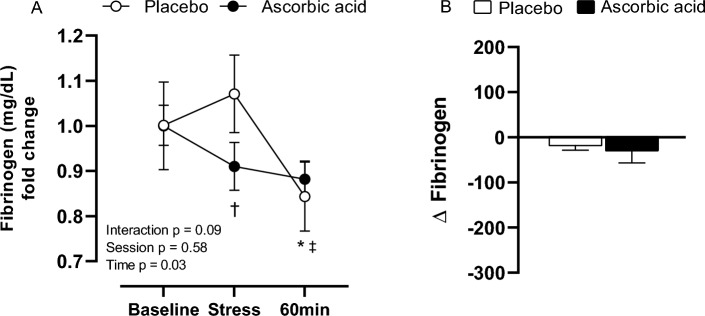
Fibrinogen concentration (**A**) and its (**B**) response to stress after placebo (PL) and ascorbic acid (AA) administration in overweight/obese men. Δ represents 60-min minus baseline. *p < 0.05 versus baseline; †*p* < 0.05 versus placebo; ‡*p* < 0.05 versus stress.

There was a significant reduction in fibrinogen concentration (baseline, 1.00 ± 0.04 mg/dL vs 60 min after stress, 0.84 ± 0.08 mg/dL, *p* = 0.04; stress, 1.07 ± 0.09 mg/dL vs 60 min after stress, 0.84 ± 0.08 mg/dL, *p* < 0.01) in the placebo session. However, no differences were observed at AA session (baseline, 1.00 ± 0.10 mg/dL; stress, 0.97 ± 0.08 mg/dL; 60 min after stress, 0.88 ± 0.04 mg/dL). During stress, fibrinogen presented significantly lower levels in the AA session when compared to placebo (PL, 1.07 ± 0.09 mg/dL vs AA, 0.97 ± 0.08 mg/dL; *p* = 0.04). Also, there were no differences in stress response (PL, Δ − 16.91 ± 40.46 mg/dL vs AA, Δ − 28.44 ± 97.17 mg/dL) (Fig. 5).

**Figure 6:**
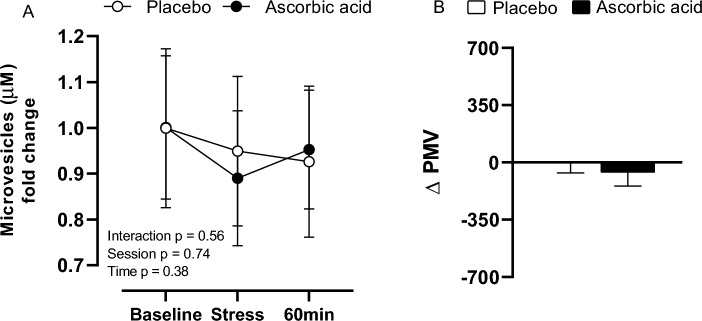
Platelet-derived microvesicles concentration (**A**) and its delta (**B**) response to stress after placebo (PL) and ascorbic acid (AA) administration in overweight/obese men. Δ represents 60-min minus baseline.

No differences were observed in PMV response to stress between sessions [PL (baseline, 1.00 ± 0.17 MV/µL; stress, 0.95 ± 0.16 MV/µL; 60 min after stress, 0.93 ± 0.16 MV/µL); AA (baseline, 1.00 ± 0.16; stress, 0.89 ± 0.15 MV/µL; 60 min after stress, 0.95 ± 0.13 MV/µL)] or in response to stress (PL, Δ 4.91 ± 237.97 MV/µL vs AA, Δ − 54.72 ± 298.68 MV/µL; *p* < 0.01) (Fig. 6).

### Nitrite measurement

**Figure 7:**
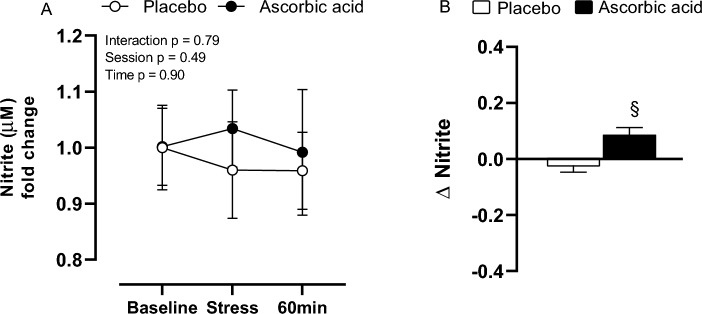
Nitrite concentration (**A**) and its delta (**B**) response to stress after placebo (PL) and ascorbic acid (AA) administration in overweight/obese men. Δ represents 60-min minus baseline. §*p* < 0.05 versus placebo.

Even though there were no differences in nitrite bioavailability between sessions [PL (baseline, 1.00 ± 0.08 µM; stress, 0.96 ± 0.08 µM; 60 min after stress, 0.95 ± 0.07 µM); AA (baseline, 1.00 ± 0.07 µM; stress, 1.03 ± 0.07 µM; 60 min after stress, 0.99 ± 0.11 µM)], the nitrite response to stress was higher in AA session when compared to PL (PL, Δ − 0.02 ± 0.08 µM vs AA, Δ 0.09 ± 0.08 µM; *p* = 0.003) (Fig. 7).

## Discussion

Novel findings from the present study are: (1) acute stress induces a procoagulant state, through reducing prothrombin time; (2) ascorbic acid prevented the procoagulant response to stress, through increasing the prothrombin time and reducing the fibrinogen concentration, especially 60 min after MS; (3) ascorbic acid increased the nitrite concentration in response to stress in overweight/obese men.

Several studies have been using stress tasks to better understand the cardiovascular pathophysiological processes^21^. A greater hemodynamic responsivity to acute stress, even when it is developed in a controlled environment (i.e., research laboratory), represents an increased risk of cardiovascular events^22^. Then, the laboratory stress tasks may help us to determine underlying mechanisms involved in cardiovascular responses or to identify preventive strategies to manage it. The modified Stroop color word test is a well-established protocol that induces psychological hemodynamic and vascular responses^20,23–25^ through increasing sympathetic drive^26^ and activating the renin angiotensin II system^27^. The present study reinforced those findings, demonstrating that stress tasks were able to similarly increase blood pressure and heart rate of overweight / obese men, even in the presence of ascorbic acid.

Previous studies have demonstrated an association between psychological stress and hypercoagulability^28^. In the present study, the stress task was able to reduce PT and aPTT in overweight and obese men, immediately after stress and 60 min after MS, providing evidence of a possible long-lasting effect of stress on hemostasis. These findings suggest a possible underlying mechanism for increased risk for acute myocardial infarction and sudden cardiac death days after stressful events^29–32^. Increased plasma levels of catecholamines and glucocorticoids seem to induce platelet activation and a higher concentration of procoagulant factors, such as tissue factor (TF), von Willebrand factor, factors VII, VIII and XII besides the fibrinogen^33,34^. Vascular injury, remodeling, or endothelial dysfunction triggers TF exposure in the subendothelial cell membrane, stimulating extrinsic and intrinsic coagulation pathways^35^.

Although stress exerts faster effects on the extrinsic coagulation pathway, it also seems to reduce fibrinogen concentration one hour after the task. Hepatic fibrinogen plays an important role in hemostasis as it is the thrombin' substrate to form fibrin polymers and blood clot^36^. High plasmatic fibrinogen levels were already demonstrated in several inflammatory and oxidant pathological conditions^36^. Also, the norepinephrine administration, usually present in acute stressful conditions, increased fibrinogen levels, d-dimer and FVIII clotting activity^37^. Then, its reduction after a stress task could mean an increased thrombin activity or even distinct plasmatic fibrinogen responses to physiological (i.e., Stroop color word task) or pharmacological (i.e., norepinephrine infusion) protocols for inducing stress.

Strategies to reduce reactive oxygen or nitrogen species (ROS-RNS) could be good alternatives to diminish the impact of daily life stressful situations on cardiovascular function, especially in populations at increased cardiometabolic risk. Ascorbic acid is a water-soluble vitamin known to neutralize ROS-RNS, and its venous administration was able to prolong the PT and reduce fibrinogen response to stress task in overweight and obese men. Previous studies published by our group^38,39^ and others^40^ reinforce that finding, demonstrating that ascorbic acid prevented redox imbalance and reduced vascular remodeling in overweight and obese men^38,39^. Also, it was already demonstrated that fibrinogen is a vulnerable target for oxidant attack^41^. Increased ROS-RNS seem to alter its primary and three-dimensional protein structure or posttranslational oxidative modifications that result in partial or complete loss of fibrinogen function and/or alternative assembly of fibrin^42^. It is believed that increased nitrite concentration, a representative of NO bioavailability, induced by ascorbic acid administration would restore the hemostatic function.

Indeed, the present study demonstrated that ascorbic acid increased the nitrite concentration despite the stress. This result was observed when the magnitude of response was analyzed, suggesting that the effects of ascorbic acid on nitrite concentration were not immediate to MS but rather delayed, possibly by the pharmacokinetics of ascorbic acid infusion^43–45^. Reductions in NO bioavailability induce hypercoagulability state in overweight or obese adults^1^. NO acts by increasing cyclic GMP levels and reducing intracellular calcium in platelets, avoiding platelet aggregation and clot formation^46^. Enhanced ROS production reduces intraplatelet NO bioavailability and its receptor activity (sGC)^47^, contributing to platelet hyperaggregability and impaired vascular function^48,49^.

No differences were observed in PMV concentration among moments and sessions. Results considering PMVs measurements are controversial. One hand, studies have demonstrated a positive correlation between PMVs release and metabolic, inflammatory, and pro-thrombotic parameters in obese subjects^50^. On the other hand, some studies demonstrated no significant difference in platelet-activation biomarkers’ expression between obese and non-obese individuals^51^. It is likely that the use of distinct superficial biomarkers for PMV identification might explain the unequal results among studies with overweight/obese adults.

### Limitations

Some limitations should be taken into consideration for our results’ interpretation. The present study did not measure redox homeostasis parameters nor catecholamines levels. However, studies from our group and others^38,40^ have shown the effects of ascorbic acid on oxidative stress after exposure to a mental stress task. Considering those aspects already shown, the present study focuses on studying whether the ascorbic acid, a well-known antioxidant, is able to blunt hemostatic responses to stress. Also, present results cannot be extrapolated to other populations, such as women. It is recognized that the risk of atherothrombotic diseases is higher in middle-aged men than women^52^. However, further studies are important to better investigate and understand the hemostatic responses to stress in age-matched women. Moreover, all the participants were young healthy overweight/obese adults, whereby the magnitude of obesity-mediated disorders is, presumably, much less aggravated than in older subjects.

## Conclusion

Our results suggest that stress exposure induces a pro-thrombotic response in overweight and obese individuals once it led to reductions in prothrombin and activated thromboplastin times. Also, ascorbic acid seems to have an anticoagulant role, increasing the prothrombin time and the nitrite concentration, whereas reducing fibrinogen concentration in response to stress.

Preventive and therapeutic strategies to reduce the impact of stress on vascular health are necessary to minimize the risk of cardiovascular morbimortality, especially in adults with overweight/obesity.

## Data Availability

The datasets used and/or analyzed during the current study are available from the corresponding author on reasonable request.
